# Different Neural Mechanisms Underlie Deficits in Mental Flexibility in Post-Traumatic Stress Disorder Compared to Mild Traumatic Brain Injury

**DOI:** 10.3389/fpsyt.2015.00170

**Published:** 2015-12-03

**Authors:** Elizabeth W. Pang

**Affiliations:** ^1^Division of Neurology, Hospital for Sick Children, Toronto, ON, Canada; ^2^Neurosciences and Mental Health, Sick Kids Research Institute, Toronto, ON, Canada; ^3^Faculty of Medicine, University of Toronto, Toronto, ON, Canada

**Keywords:** mental flexibility, executive function, set-shifting, post-traumatic stress disorder, traumatic brain injury, concussion, magnetoencephalography, functional connectivity

## Abstract

Mental flexibility is a core executive function that underlies the ability to adapt to changing situations and respond to new information. Individuals with post-traumatic stress disorder (PTSD) and mild traumatic brain injury (mTBI) complain of a number of executive function difficulties, one of which is mental *in*flexibility or an inability to switch between concepts. While the behavioral presentation of mental inflexibility is similar in those with PTSD or mTBI, we hypothesized that the differences in their etiology would manifest as differences in their underlying brain processing. The neural substrates of mental flexibility have been examined with a number of neuroimaging modalities. Functional magnetic resonance imaging has elucidated the brain regions involved, whereas electroencephalography has been applied to understand the timing of the brain activations. Magnetoencephalography, with its high temporal and spatial resolution, has more recently been used to delineate the spatiotemporal progression of brain processes involved in mental flexibility and has been applied to the study of clinical populations. In a number of separate studies, our group has compared the source localization and brain connectivity during a mental flexibility set-shifting task in a group of soldiers with PTSD and civilians with an acute mTBI. In this article, we review the results from these studies and integrate the data between groups to compare and contrast differences in behavioral, neural, and connectivity findings. We show that the different etiologies of PTSD and mTBI are expressed as distinct neural profiles for mental flexibility that differentiate the groups despite their similar clinical presentations.

## Introduction

Understanding the nature of cognitive dysfunction in different clinical conditions is essential for prescribing appropriate therapies and for developing new targeted interventions. Both post-traumatic stress disorder (PTSD) and a mild traumatic brain injury (mTBI) are considered acquired brain injuries, and although they can occur on their own, they often co-exist or present with similar symptoms (1). This overlap is particularly prevalent among military populations (2, 3) and presents a challenge for differential diagnosis. Despite good advances in our understanding of PTSD and mTBI, there remains a great need to continue to identify specific neural mechanisms impacted by either condition, and to explore how dysfunction within these neural mechanisms contribute to, and/or exacerbate, clinical symptomology. This focus, while narrow, is one way to probe deeper into the brain mechanisms that are injured to gain an understanding of how the injury may manifest itself in PTSD or mTBI.

Over the years, our laboratory has used magnetoencephalography (MEG) brain recordings to examine cognitive function in a number of different clinical disorders [for reviews, see Ref. (4, 5)]. Our choice of this modality is based on the advantage that MEG offers both high temporal resolution (in the milliseconds) and very good spatial resolution (in the millimeters). As such, MEG is the ideal choice for examining fast-paced cognitive processes [for a review, see Ref. (6)]. Recently, our group has applied our MEG methods to the study of PTSD and mTBI, exploring the neural underpinnings of various cognitive deficits and, in this article, the results from a subset of these studies that investigated mental flexibility using a set-shifting task are discussed.

Dolan et al. (7) presented a review that compared neuroimaging studies in PTSD and mTBI. They summarized differences and similarities in the presentation of neuropsychological deficits between these two groups. Some items on their list clearly differentiated between PTSD and mTBI (for example, attention, inhibition, and working memory), whereas other items showed mild deficits for both groups (for example, set-shifting). From this summary, one would presume that the brain processes underlying the domains that clearly differentiate the groups would be different while the brain processes underlying domains that have similar presentations would be more similar. To our surprise, our investigation into the neural correlates of set-shifting in PTSD and mTBI showed striking differences in brain function, despite similar behavioral performances. In this perspective article, we collate our MEG data on mental flexibility in individuals with PTSD and mTBI, and reconcile the MEG findings with what is known about the mechanisms of injury. In describing this focused body of work, we hope to demonstrate the power of high spatiotemporal resolution neuroimaging in detecting brain differences despite phenotypically similar presentations. This kind of neuroimaging holds potential for developing new methods for differential diagnosis and offers new avenues for designing individually tailored rehabilitation.

## Cognitive Executive Functions

Cognitive executive functions refer to those abilities required to achieve independent goal-oriented behavior (8). Cognitive dysfunction encompasses a broad domain and the nature of the cognitive sequelae can result from a number of different factors. Although not exclusively limited to the frontal lobes, the frontal lobes are thought to carry out the majority of processes involved in cognitive executive functions (9).

In a commonly accepted model of the neural substrates of cognitive executive function, the frontal lobes are divided into anatomically discrete categories that subserve distinct functions. These divisions are as follows: the dorsomedial cortex is required for activation and initiation; the lateral frontal cortex for organizing, planning, reasoning, set-shifting, and monitoring; the ventral–medial/orbital cortex for emotional and behavioral regulation, including inhibition, impulsivity, etc.; and the frontopolar cortex for integration and meta-cognitive functions (10). Thus, using this model, one could postulate that if one knew the mechanism of injury and brain site of injury then one could predict which cognitive deficit would ensue.

### Executive Dysfunction in PTSD

The DSM-V (11) criteria for diagnosing PTSD include a history of exposure to a traumatic event with the resultant response involving memory intrusions, trigger avoidance, negative changes in cognition and mood, and alterations in arousal and reactivity. Although the clinical presentation varies, cognitive symptoms are commonly reported, and include impaired concentration, affect, and increased impulsivity. This combination of cognitive-emotional and cognitive-behavioral difficulties results in significant distress as well as impairments in functioning [for review, see Ref. (12)].

Much of the neuropsychological research on PTSD has focused on learning and memory; however, there is increasing evidence suggesting that executive dysfunction plays an important role. A recent review systematically explored this topic and collated studies that directly measured executive functioning in PTSD. The results showed that, in general, the PTSD group did more poorly on both memory and cognitive flexibility, although the precise nature of the deficit was heterogeneous (13). Taking this one step further, another review (14) collated factors that may contribute to PTSD susceptibility or provide disease resilience. The authors proposed that difficulties with executive functions in PTSD are not separate symptoms, but these difficulties underlie and sustain the clinical symptoms of hyperarousal, hypervigilance, and intrusive memories.

It has been proposed (15) that the mechanism of action of PTSD stems from inadequate modulation of the limbic systems by medial prefrontal cortices, suggesting deficits in both limbic systems and medial prefrontal cortex. Referring back to the model of the neural substrates of cognitive executive functions (10) described above (see Cognitive Executive Functions), deficits in the medial prefrontal cortex function would impact task activation and initiation. This impact would fit well with the commonly seen PTSD symptoms of depression, anxiety, and lethargy but would not support an impact on executive dysfunction and mental inflexibility. However, our MEG data (described in the Section “MEG Studies of Mental Flexibility in PTSD”) raise the possibility of a hybrid model, whereby the limbic systems are inadequately modulated, not because the medial prefrontal cortices are deficit, but because the limbic response is so overwhelming that they interfere with the normal neural processing sequence. This will be further explored in the Section “MEG Studies of Mental Flexibility in PTSD” below.

### Executive Dysfunction in mTBI

A mild traumatic brain injury is defined as an insult to the brain from an external force but can occur *without* direct impact to the head. The mechanism of injury results from biomechanical forces that undergo rapid acceleration and deceleration in both a linear and rotational direction. Linear forces likely cause contusions in the frontal and temporal regions, whereas rotational forces likely cause diffuse axonal injury to the brain’s white matter tracts (16). While the impact of these translational and rotational forces cannot be seen on traditional structural MRI imaging in mild TBI (17, 18), new diffusion tensor imaging methods have shown correlations between executive dysfunction and mTBI-related axonal injury in the dorsolateral prefrontal cortices (19), as well as frontal and temporal white matter damage that is related to the degree of cognitive dysfunction (20).

There are numerous reviews describing the impact of mTBI on cognitive dysfunctions, but a recent “review of the reviews” (21) reported that, overall, cognitive dysfunctions exist; however, the magnitude of the effect size for each cognitive domain was variable. Furthermore, there is evidence demonstrating that while all individuals who suffer a concussion demonstrate measurable neurocognitive abnormalities at 15 min post-injury, these symptoms resolve within 48 h for the majority of sufferers (22). However, a percentage, as high as 15% (23), of individuals with a mild TBI go on to have persistent symptoms and these symptoms often involve a number of cognitive complaints and dysfunctions (16).

Referring again to the neural substrates model of cognitive executive functions (10), a traumatic brain injury, with its known frontal/temporal damage, should demonstrate deficits in functions subsumed in lateral cortices (24). These functions include planning, organizing, reasoning, set-shifting, and monitoring. Thus, this model would predict that a traumatic brain injury would have direct impact on set-shifting ability.

## Assessment of Mental Flexibility

Mental flexibility is a core executive function that allows individuals to update their cognitive strategies in the face of changing goals or environments and is essential to adaptive behavior and learning. The neuropsychological evidence for a specific role of mental *in*flexibility in the pathophysiology of both PTSD and mTBI has been equivocal, and it has been suggested that while both groups show mild deficits, there are no differentiable effects in this domain (7). On the other hand, the neural model of executive functions (24), described above, would postulate that the two groups present differently on this task. To explore these opposing views, a neuroimaging modality with high temporal and spatial resolution, such as MEG, may offer insight. Before we present the neuroimaging literature, we will first present the standard assessments used to measure mental flexibility.

In standardized neuropsychological assessments, mental flexibility is assessed with the Stroop test (25), the trail making test (26, 27), and the Wisconsin card sorting task (WCST) (28). An alternative to the WCST that places fewer cognitive demands on participants is the attentional set-shifting, intra-extra dimensional set shift (IED) test (29), from the Cambridge Neuropsychological Test Automated Battery (CANTAB^®^, Cambridge Cognition). We have operationalized the IED set-shifting test for use with MEG neuroimaging. We applied this to individuals with PTSD and mTBI, and we will describe our findings in Sections “MEG Studies of Mental Flexibility in PTSD” and “MEG Studies of Mental Flexibility in mTBI” below.

## Neuroimaging Studies of Mental Flexibility

Functional magnetic resonance imaging (fMRI) studies, using the Wisconsin card sorting test, have identified neural areas involved in mental flexibility processing, which include a distributed network involving prefrontal and frontal cortical regions [e.g., Ref. (30–33)], as well as associated posterior cortical regions [e.g., Ref. (34–36)]. While fMRI and PET studies have been invaluable in localizing brain regions involved in set-shifting, these modalities cannot capture the millisecond timing, and the fine temporal characteristics, of this fast-paced neurocognitive process. Thus, this is an opportunity where MEG recordings may shed light.

### MEG Studies of Mental Flexibility

Magnetoencephalography has been used to examine the spatiotemporal dynamics of mental flexibility processes in typical adults. Using both traditional (37) and modified (38, 39) versions of the WCST paradigm, shifting processes were identified in dorsolateral prefrontal cortex (dlPFC; Brodmann area 9) (37) including the superior frontal gyrus (38), as well as the inferior frontal gyrus (BA 45, 47/12), the anterior cingulate, and supramarginal gyrus (BA 40) (38). These findings fit well the neural model of frontal lobe executive functions (10).

Using the simple intra-extra dimensional set-shift paradigm, our group (40) found that easy shifts activated right inferior frontal gyrus (BA 47) and bilateral dorsolateral prefrontal cortices (right BA 9, left BA 10/11), while more difficult shifts, in addition to those listed above, also recruited right superior frontal gyrus (BA 8/10) and left inferior frontal gyrus (BA 44). Furthermore, bilateral parietal areas were activated in both easy and hard conditions. Together, these MEG studies established that a widespread network of frontal and parietal regions is involved in set-shifting in control adults, and these results serve as a baseline for comparison with clinical groups.

## Neuroimaging Studies of Mental Flexibility in PTSD

Early behavioral studies examining PTSD and mental flexibility did not find group differences in performance (41, 42). However, there have been three neuroimaging studies (two fMRI and one MEG) looking at mental flexibility in PTSD, and these show differences suggesting that group differences may be subtle. An fMRI study reported that individuals with PTSD failed to activate the right insula when performing an affective set-shifting task (43) while elite military warriors without PTSD showed increased right anterior insula activation, perhaps reflecting their ability to perform well in highly stressed military situations (44).

### MEG Studies of Mental Flexibility in PTSD

We conducted an MEG study where soldiers with and without PTSD were compared on a battery of executive functions task, including a set-shifting task (45, 46). We (46) found that the control soldiers showed a sequence of activations that looked comparable to adult non-military controls (40), involving dorsolateral frontal cortex, insula, and posterior parietal cortices. On the other hand, the soldiers with PTSD showed these same activations; however, these activations were interrupted by activations in paralimbic regions, specifically the posterior cingulate, parahippocampal gyri, and regions in the temporal lobes.

These findings bring to mind the models of PTSD that suggest that the dysfunctional neurocircuitry seen in PTSD is driven by hyper-reactive limbic areas that are not appropriately modulated by prefrontal cortical control regions (15, 47). Specifically, we also found a dissociation of the response in the paralimbic structures – that is, we found increased activation in the cingulate and parahippocampal cortex in the group with PTSD, while the medial prefrontal cortex and the insula were significantly less active. These findings fit with the model of PTSD and suggest that while cortical regions are active and function normally, it is the hyperactive limbic system which interrupts and disrupts their function, with the result being a less efficient pathway of neural activation, and ultimately, a negative impact on function.

Furthermore, our results corroborate the fMRI studies by Simmons and colleagues (43, 44), which showed that individuals with PTSD failed to adequately activate the right insula when performing a set-shifting task, and the authors proposed that the right insula was a key region for resilience against PTSD and maintenance of performance during stressful situations. We specifically tested this hypothesis by re-constructing time courses of activation in the right insula, and we saw a significant difference with a greater and earlier activation in the right insula in the control soldiers compared to the soldiers with PTSD. This would suggest that the control soldiers specifically recruited the insula, and there is not simply widespread, greater activation in the controls. This may be one mechanism that impedes performance on a set-shifting task in PTSD, possibly contributing to difficulties with mental flexibility, which may underlie deficits in other cognitive executive functions.

Finally, we submitted our data to MEG connectivity analyses and found that over all, there were significant large-scale increases in connectivity in the theta frequency band in the PTSD group compared to controls. This hyperconnectivity was concentrated in networks in the right parietal cortex, and the strength of this hyperconnectivity correlated with increased scores on measures of attention, depression, and anxiety, as well as decreases in performance on the mental flexibility task. Taken together, these data suggest that brain network hyperconnectivity may underlie the mental flexibility difficulties seen in PTSD, and this hyperconnectivity may also play a more general role in the other cognitive sequelae seen in PTSD (45).

Recalling Stuss’ model (10) of the neural substrates underlying frontal lobe executive functions, these MEG data suggest that the mild deficits in mental flexibility seen in PTSD are not due to frontal dysfunction of set-shifting systems, but due to excessive limbic activation that blocks the normal sequence of activations and dissipates processing power via hyperconnectivity in key processing nodes.

## Neuroimaging Studies of Mental Flexibility in mTBI

A review of the fMRI literature in mTBI (48) found that in the decade between 1999 and 2009, there were fewer than 20 articles investigating cognitive functioning after an mTBI; these focused on executive functions, including working memory and attention and resting state functional connectivity. The authors concluded that while the studies thus far have enhanced our understanding of the impact of an mTBI on executive functions, working memory, attention, and resting state functional connectivity results have not been definitive, and there is a need for continued investigations of task-related activations within specific brain networks [review by McDonald et al. (48)].

### MEG Studies of Mental Flexibility in mTBI

There are even fewer MEG studies in mTBI, and these have focused on exploring brain dynamics with resting state functional connectivity analyses. These studies show that MEG functional connectivity can identify sites of brain injury (49, 50), differentiate individuals with mTBI from controls (51), and correlate cognitive recovery (52) and neuropsychological assessments (53) with decreases in functional connectivity.

To the best of our knowledge, there has only been one MEG study examining the impact of an mTBI on mental flexibility. In parallel with the PTSD study described above in the Section “MEG Studies of Mental Flexibility in PTSD,” we acquired MEG data in adult men with a mild TBI recruited from the emergency room at a local trauma unit and compared their brain activations, during a mental flexibility set-shifting task, to a group of adult controls. While accuracy was similar between groups, reaction time was significantly slower in the mTBI group, and their sequence of brain activations was disorganized and recruited additional regions not typically involved in set-shifting. We took this as evidence that individuals with mTBI experience slower and more effortful processing, requiring additional compensatory regions to come on-line to accomplish the task (54). Submission of this dataset to connectivity analyses would be an interesting next step, which may offer additional insight as to which brain regions become less connected and how compensatory mechanisms are working.

Referring again to the neural model of executive functions (10), a traumatic brain injury, with its known frontal/temporal damage, should demonstrate deficits in functions subsumed in lateral cortices (24). Our finding of specific neural differences on a set-shifting task supports this model. It should be noted, however, that the participants in our MEG study (54) were recruited and tested within 2 months of their injury although on measures of health status, symptom severity, and symptom count, the mTBI group reported to be significantly more affected than controls. Since the literature (22) shows that the majority of symptoms resolve within days to weeks, it is likely that our study tapped early manifestations of the chronic aspects of the cognitive deficits seen in post-concussive syndrome, although this requires further empirical testing.

## General Discussion

The neural model of frontal lobe executive functions (10) would predict that, given the different mechanisms of injury involved in PTSD and mTBI, these conditions would perform differently on tasks of mental flexibility. In our studies, while the behavioral and neuropsychological assessments of mental flexibility looked very similar between these groups, our MEG data showed stark differences that clearly differentiate the two groups. The PTSD group showed abnormal activations in paralimbic systems that acted as an obstruction to normal cognitive processing, while the mTBI group showed reduced cognitive processing ability as evidenced by disorganized and delayed brain activations. These findings not only fit known neuroanatomical models of frontal lobe cognitive functions but also indicate that approaches to rehabilitation and therapy need to consider that different neural mechanisms are at play in these disorders. This would suggest that interventions tailored toward addressing the specific dysfunctional mechanism would offer a more effective treatment and long-term outcome. The impact of these studies and their implications for addressing these disorders underline the value of the high temporal and spatial resolution of MEG and its potential utility to change the way we diagnose and treat PTSD and mTBI.

## Conflict of Interest Statement

The author declares that the research was conducted in the absence of any commercial or financial relationships that could be construed as a potential conflict of interest.
